# The role of magnesium in pancreatic beta-cell function and homeostasis

**DOI:** 10.3389/fnut.2024.1458700

**Published:** 2024-09-25

**Authors:** Nuraly S. Akimbekov, Seval Ozkan Coban, Azeddine Atfi, Mohammed S. Razzaque

**Affiliations:** ^1^Scientific-Practical Center, West Kazakhstan Marat Ospanov Medical University, Aktobe, Kazakhstan; ^2^Sustainability of Ecology and Bioresources, Al-Farabi Kazakh National University, Almaty, Kazakhstan; ^3^Department of Medical Education, School of Medicine, University of Texas (UTRGV), Edinburg, TX, United States; ^4^Department of Biochemistry and Molecular Biology, Massey Cancer Center, Virginia Commonwealth University, Richmond, VA, United States

**Keywords:** magnesium, diabetes, insulin, glucose, β-cells

## Abstract

Magnesium plays an essential role in glucose utilization and insulin signaling. Recent advances have revealed a greater prevalence of hypomagnesemia in general, and low intracellular magnesium levels in individuals with diabetes contribute to β-cell dysfunction and insulin resistance. This article describes the documented effects of magnesium on various aspects of β-cells and glucose homeostasis. Studies have demonstrated that magnesium deficiency is associated with reduced pancreatic β-cell activity and increased insulin resistance in patients with type 2 diabetes. Additionally, magnesium is involved in many cellular events, including energy homeostasis, protein synthesis, and DNA stability. Furthermore, magnesium is critical for proper glucose utilization and insulin signaling, and magnesium deficiency can lead to the dysregulation of ATP-sensitive potassium (KATP) channels in pancreatic β-cells, impairing insulin secretion. Therefore, maintaining adequate magnesium levels is crucial for maintaining overall health and preventing of metabolic disorders such as type 2 diabetes.

## Introduction

Magnesium is one of the most abundant minerals in the human body. It is essential for many biochemical reactions and is indispensable for preserving normal cellular and organ functions, ranging from cellular energy metabolism to skeletal mineralization (1, 2). Maintaining an optimal magnesium balance is essential, as any deviation may lead to dysregulated glucose turnover. Magnesium plays a vital role in regulating glucose homeostasis by modulating the functions of pancreatic β-cells. In type 2 diabetes, magnesium deficiency can increase insulin resistance, and insulin resistance can also contribute to magnesium deficiency. Intracellular free magnesium levels are lower in patients with type 2 diabetes than in nondiabetic individuals. Intracellular magnesium concentrations are essential for optimal insulin receptor bioactivity and downstream signaling events in target cells. Low magnesium levels contribute to impaired tyrosine kinase activities of insulin receptors, causing altered cellular glucose utilization and eventual insulin resistance. An increased incidence of hypomagnesemia has been reported in patients with type 2 diabetes. Numerous clinical studies have documented the benefits of magnesium supplementation on the metabolic profiles of patients with diabetes. Based on the existing evidence, the potential effects of magnesium on various cellular aspects of glucose homeostasis are briefly discussed.

Pancreatic β-cells are clumped together in groups called islets. β-cells generate insulin to regulate blood glucose homeostasis. In response to elevated blood glucose levels, β-cells secrete increasing amounts of insulin to increase the cellular uptake of glucose from the blood to store it as glycogen, primarily in liver and muscle cells. Depending on the body's needs, stored glycogen can be broken down into glucose for further use. β-cells can also release the hormone amylin, which reduces the amount of glucose entering the bloodstream to decrease blood glucose elevation after meals. In certain disease conditions, such as type 1 diabetes mellitus, when pancreatic β-cells are damaged or destroyed, insulin synthesis is impaired, resulting in elevated blood glucose levels. In type 2 diabetes, despite pancreatic β-cells producing enough or even excess insulin, the body develops insulin resistance, where effector tissues (mainly adipose tissue, liver, and muscle) are unable to convert bloodstream glucose into glycogen. Insulin resistance reduces glucose utilization, causing a compensatory increase in the production of insulin by β-cells. In situations where blood glucose levels are consistently high, physiological communication or sensing between the glucose load and the pancreatic β-cell response is lost, and exhausted β-cells gradually lose their ability to generate the required amounts of insulin to control the blood glucose balance. Chronic insulin resistance can eventually lead to type 2 diabetes mellitus (3). In addition to β-cells, the islets also contain pancreatic α-cells, which store and release glucagon to maintain optimal blood glucose balance by stimulating liver and muscle cells to convert glycogen back into glucose for energy utilization when necessary. Fine-tuning between insulin and glucagon stabilizes the sugar balance. The development of effective nutritional intervention methods for maintaining pancreatic β-cell functions is an intense area of research for diseases related to increased glucose burden. Magnesium is one of the most important players in β-cells and is necessary for adequate glucose utilization and optimal insulin signaling. Importantly, studies have shown a greater prevalence of hypomagnesemia and lower intracellular concentrations of magnesium in patients with diabetes (4). Dysregulation of the concentration of cellular magnesium, which acts as a second messenger for insulin bioactivity, is likely to contribute to insulin resistance (5). This brief article discusses the cellular effects of magnesium on various aspects of glucose homeostasis.

## Magnesium

In humans, magnesium is the second most abundant intracellular cation after potassium. Magnesium is also the fourth most common mineral in the human body (after calcium, potassium, and sodium) (1, 6). According to enzymatic databases, magnesium acts as a cofactor or activator for more than 800 enzymes (6). Most magnesium is present in bone and teeth (~60%), while its concentrations in intracellular compartments and extracellular fluids constitute ~40% and <1%, respectively (2, 6–9). The normal blood range of magnesium is 1.8–2.3 mg/dl (0.75–0.95 mmol/L). However, studies have shown that serum magnesium concentrations <0.85 mmol/L are associated with increased health risks; hence, it is recommended that the lower limit of the reference range be increased to 0.85 mmol/L (2.07 mg/dL) (10). Approximately 0.3% of total body magnesium is present in blood, and consequently, the serum level of magnesium is not a good predictor of intracellular magnesium content or total body magnesium content (1, 6). The recommended dietary allowance (RDA) for magnesium is 420 mg/day for men and 320 mg/day for women. Studies in Europe and the U.S. have shown that Western diets have low magnesium content; magnesium intake in the U.S. has decreased from ~500 mg/day to 175–225 mg/day in the last 100 years (11).

The magnesium balance is controlled by delicate organ crosstalk and is regulated by intestinal absorption and renal reabsorption (6). In addition to passive paracellular intestinal absorption, a small fraction of magnesium is transported by transient receptor potential melastatin (TRPM) subfamily ion channels (TRPM6 and TRPM7). Reduced consumption of magnesium from food or drinking water, increased renal loss of magnesium, and chronic use of certain medications (including bumetanide, furosemide, and ethacrynic acid) can induce magnesium inadequacy, including hypomagnesemia (7, 12, 13). Certain nephrotoxic drugs, including cyclosporine, cisplatin, and tacrolimus, can also increase urinary magnesium wasting (14). In addition, chronic intestinal diseases, including Crohn's disease, ulcerative colitis, celiac disease, and Whipple's disease, can impair magnesium absorption (11). Skeletal magnesium is not easily exchanged, and any rapid need for magnesium is contributed by the magnesium present in the intracellular compartment.

Metformin, a commonly used drug to treat type 2 diabetes, can cause hypomagnesemia due to gastrointestinal wasting of magnesium (13) and may be one of the reasons for the increased incidence of magnesium inadequacy among patients with type 2 diabetes. An inverse relationship between magnesium consumption and the risk of diabetes has been reported in a large cohort of 37,309 participants (15). A prospective Black Women's Health Study cohort with 41,186 participants revealed that a diet with high magnesium (particularly whole grains) can substantially lower the risk of type 2 diabetes (16). Magnesium supplementation can improve the ability of pancreatic β-cells to secrete insulin and compensate for variations in insulin sensitivity in nondiabetic individuals to regulate glucose homeostasis (17).

## Magnesium and pancreatic β-cell function

**Figure 1:**
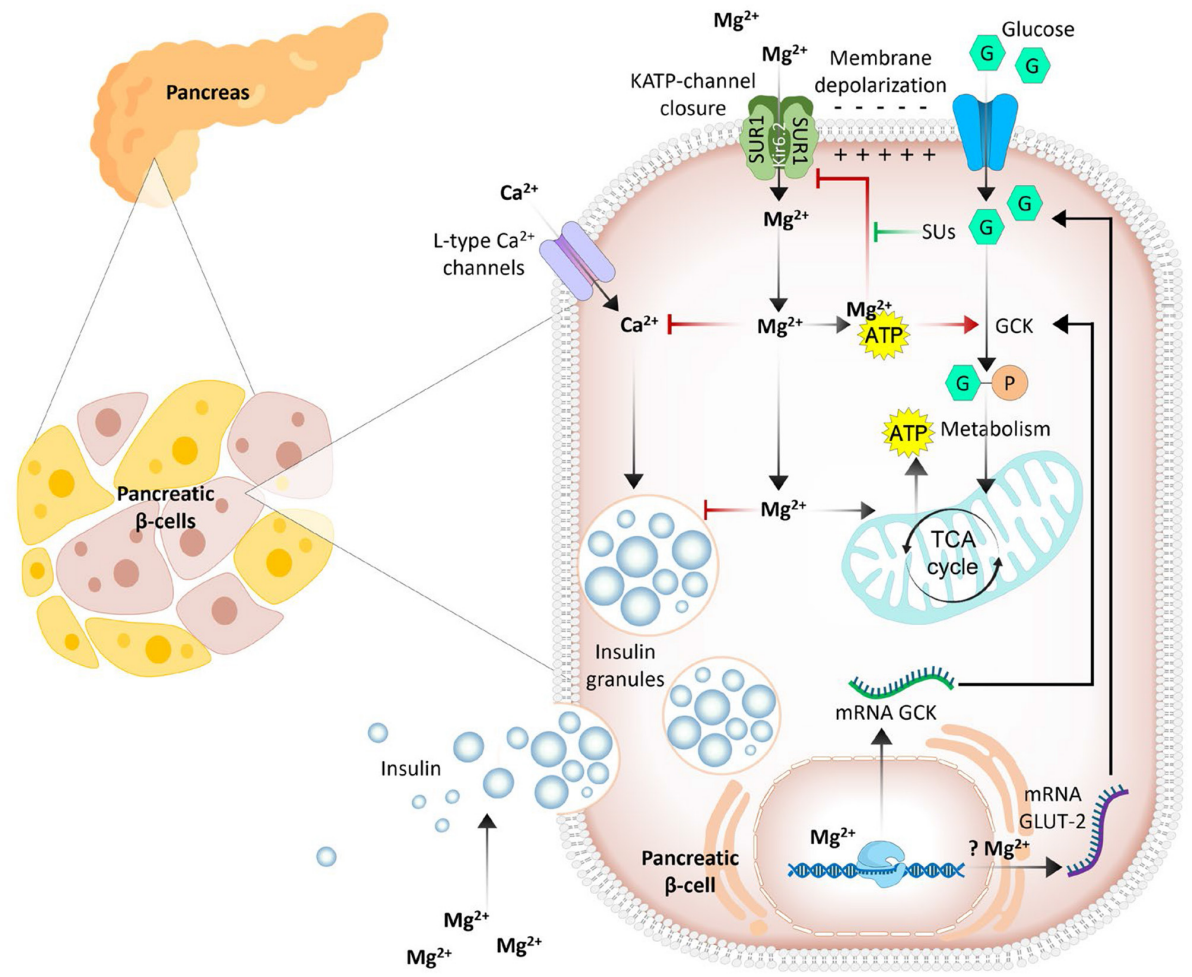
Simplified outlines of the complex dual role of magnesium in insulin secretion from pancreatic β-cells. Intracellular Mg^2+^ enhances the activity of key enzymes [glucokinase (GCK), phosphofructokinase, pyruvate kinase, and Krebs cycle enzymes], and elevated enzyme activity increases ATP levels; increased ATP inhibits ATP-dependent potassium (KATP) channels. This leads to cell membrane depolarization and Ca^2+^ influx through L-type channels. In addition, Mg^2+^ competitively inhibits L-type Ca^2+^ channels, potentially reducing insulin release. Mg^2+^ may also alter the expression of GLUT2 and L-type Ca^2+^ channel genes (7, 34).

Magnesium deficiency is associated with pancreatic β-cell dysfunction and insulin resistance, culminating in an increased risk of developing type 2 diabetes and metabolic syndrome (7, 18). An inadequate cellular magnesium balance may promote insulin resistance and alter cellular glucose influx by modulating the functions of glucokinase (5, 18). Glucokinase apparently acts as a glucose sensor in β-cells and controls the rate of cellular glucose entry (19). Magnesium plays a role in the mitochondrial synthesis of adenosine triphosphate (ATP) to form the Mg-ATP complex and can regulate glucokinase functions by acting as a cofactor (for Mg-ATP) (20). Furthermore, the ATP-sensitive potassium channel KATP (consisting of the Kir6.2 and SUR1 subunits) promotes insulin secretion by pancreatic β-cells (20). In magnesium deficiency, reduced intracellular levels of the Mg-ATP complex inhibit the opening of KATP channels and delay insulin responses to glucose. The Mg-ATP complex also activates phosphoglucomutase, an enzyme that plays a role in glucose metabolism in adipose tissue (21). In addition, cell signaling utilizes Mg-ATP for protein phosphorylation and the activation of cyclic adenosine monophosphate (cAMP), which is involved in numerous biochemical processes, including the amplification of insulin secretion in β-cells (Figure 1) (22, 23). cAMP also plays a role in regulating glucagon secretion from α-cells in the pancreas to fine-tune glucose homeostasis (23).

An adequate intracellular magnesium concentration is also critical for the phosphorylation of insulin receptors, and dysregulation of magnesium balance can induce peripheral insulin resistance, partly by desensitizing insulin receptors (18). Moreover, the affinity of insulin receptor tyrosine kinase for Mg-ATP increases when the free magnesium concentration increases, whereas the affinity of insulin receptor tyrosine kinase for free magnesium increases when the Mg-ATP concentration increases (18), implying that magnesium inadequacy could impact kinase activities to impair insulin receptor signaling and ultimately affect peripheral insulin sensitivity. Insulin can modulate the shift of magnesium from the extracellular to the intracellular compartment. The intracellular level of magnesium is tightly controlled by a few factors, including insulin (24). For example, when uterine smooth muscle cells were treated with insulin, a net intracellular surge of magnesium and potassium was detected, leading the authors to propose that insulin, after interacting with its cell surface receptors, could affect an ATPase pump to increase the cellular entry of magnesium and potassium (24). In a similar study with human erythrocytes collected from nondiabetic healthy individuals, after the ingestion of glucose, an insulin-induced shift of magnesium from the extracellular to the intracellular compartment was detected, which was associated with a reduced plasma magnesium level and a transition elevation of magnesium content in erythrocytes (25). Reverse changes in magnesium distribution were noted in the plasma and erythrocyte compartments during the euglycemic hyperinsulinemic glucose clamp (25). *In vitro* studies have suggested that in magnesium-deficient adipocytes, insulin-dependent glucose uptake is reduced by approximately 50% compared with that in control adipocytes exposed to magnesium-containing media (26). Magnesium supplementation increases insulin sensitivity and reduces glucose levels in individuals with or without diabetes (27, 28). Notably, the prevalence of hypomagnesemia in individuals with type 2 diabetes is approximately tenfold greater than that in the healthy population (29–32).

**Figure 2:**
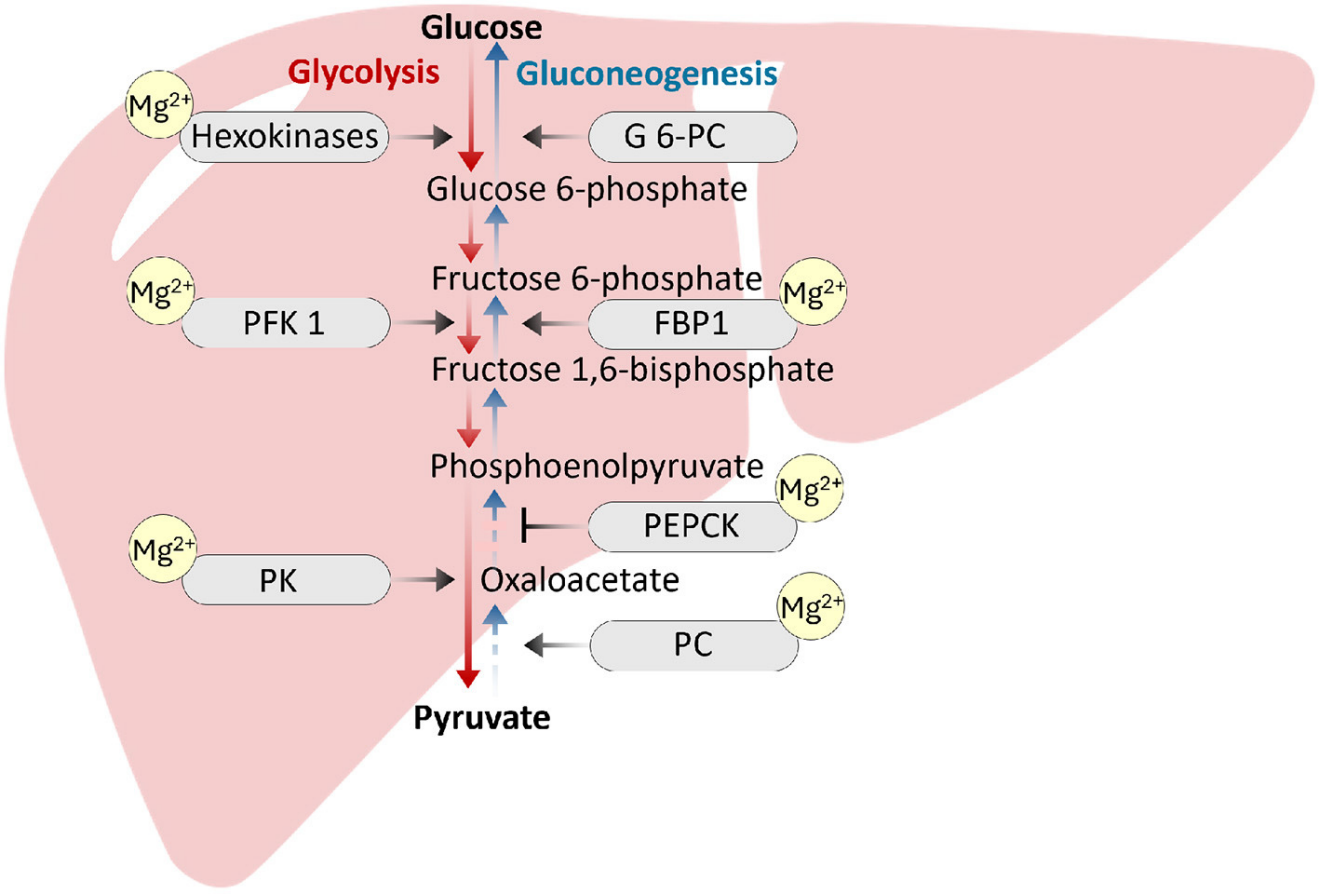
Magnesium acts as a cofactor for the functionality of rate-limiting enzymes, including phosphoenolpyruvate carboxykinase (PEPCK), fructose-1,6-bisphosphatase (FBP1) and pyruvate carboxylase (PC), during gluconeogenesis. Magnesium is also an important regulator of enzymes involved in the glycolysis pathway, including hexokinase, phosphofructokinase (PFK1), and pyruvate kinase (PK). Magnesium inadequacy can impair the enzymatic activities to affect glucose homeostasis (46–48).

Magnesium is also an important regulator of enzymes involved in glycolysis and the Krebs cycle, as documented through the ability of the Mg-ATP complex to influence the activities of several glycolytic enzymes, including hexokinase, phosphofructokinase, phosphoglycerate kinase, and pyruvate kinase, presumably by acting as cofactors for these enzymes (33). Magnesium also plays a critical role as a cofactor for the rate-limiting enzymes of gluconeogenesis, a process of generating glucose from noncarbohydrate molecules such as fatty acids and glucogenic amino acids. Magnesium acts as a cofactor for the functionality of rate-limiting enzymes, including phosphoenolpyruvate carboxy kinase (PEPCK), fructose-1,6-bisphosphatase and pyruvate carboxylase, during gluconeogenesis (Figure 2). Studies have shown that magnesium deficiency is strongly associated with increased PEPCK activity, resulting in increased gluconeogenesis in the liver (34). An association between genetic variations in specific magnesium channels or transporters and a greater risk of type 2 diabetes has also been reported (20). For example, a single nucleotide polymorphism in the TRPM6 and solute carrier family 41 member 1 (SLC41A1) genes is associated with an increased risk of type 2 diabetes (20). Additionally, genetic changes in pancreatic β-cell KATP channels can play a key role in impairing insulin secretion and the eventual development of type 2 diabetes (35). Similarly, the role of NIPA-like domain containing 1 (NIPAL1), a magnesium influx transporter, in pancreatic β-cell function and insulin secretion has been studied (36). The investigators reported that NIPAL1 expression is a magnesium-dependent process and is localized to the Golgi in β-cells. NIPAL1 knockdown decreases basal insulin secretion and total insulin content, whereas NIPAL1 overexpression increases total insulin content. These results suggest that NIPAL1 plays a crucial role in insulin production and secretion, particularly under hypomagnesemia conditions, which are common in type 2 diabetes patients.

Hyperglycemia and hyperinsulinemia can increase urinary magnesium loss and contribute to magnesium inadequacy (37). In animals with diabetes, magnesium supplementation has been shown to increase insulin sensitivity and decrease insulin resistance (38). Similar benefits of magnesium supplementation in reducing insulin resistance have been reported in individuals with hypomagnesemia presenting with insulin resistance (39). Increased serum magnesium is related to increased insulin sensitivity (40). A meta-analysis reported a linear relationship between dietary magnesium intake and metabolic syndrome, with an increase of 150 mg/day leading to a 12% reduction in the risk of metabolic syndrome (41). Another study involving 4,637 U.S. adults aged 18–30 years reported that the risk of metabolic syndrome was reduced by 31% among individuals in the highest quartile of dietary magnesium consumption (42). It is important to maintain an optimal magnesium balance, along with other minerals and vitamins, to support normal physiological functions, including metabolic health (43–45).

## Conclusions

**Figure 3:**
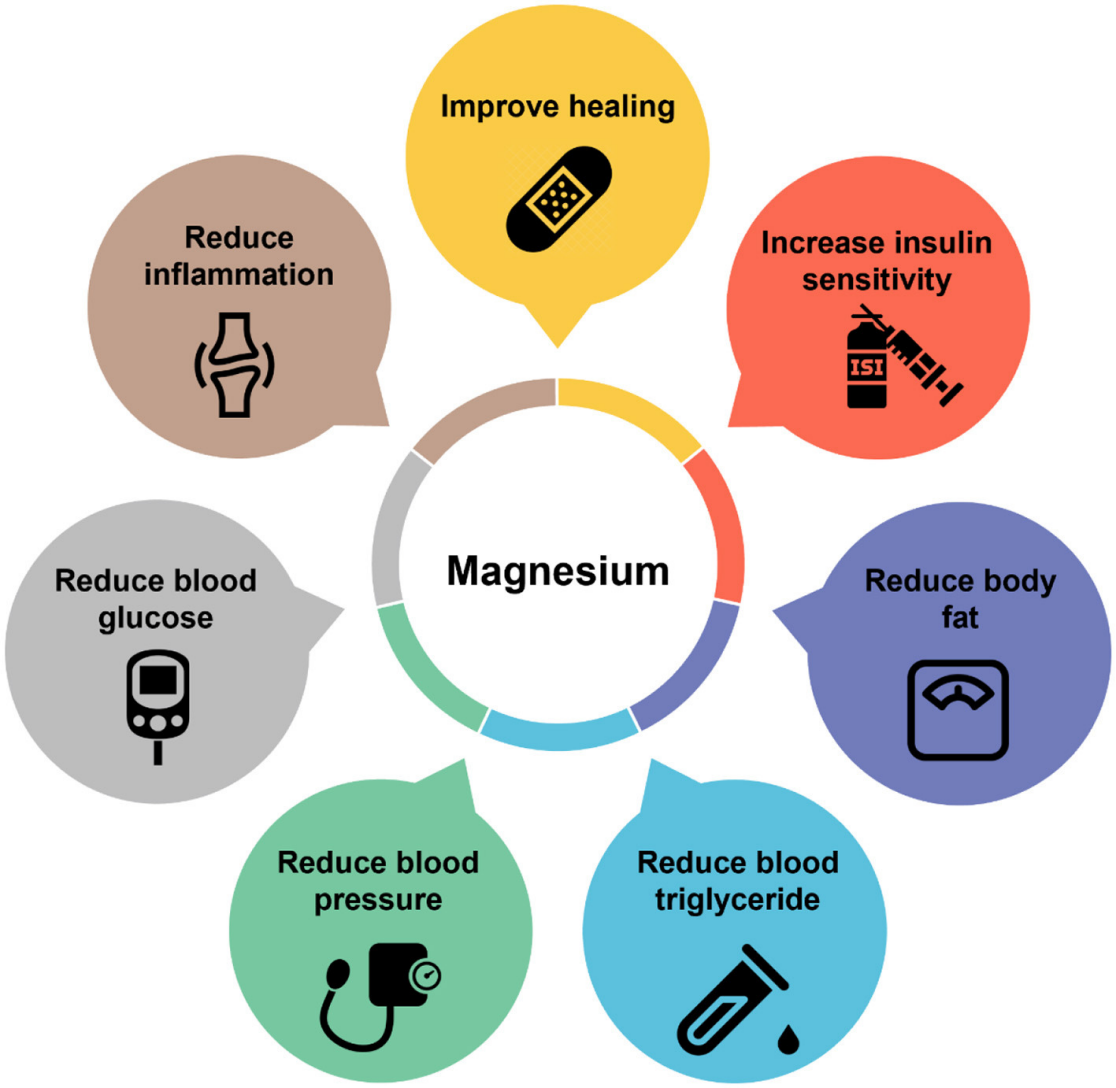
Partial list of the wide range of health-promoting functions of magnesium.

Adequate magnesium balance is necessary for a wide range of functions, including pancreatic β-cell function and optimal insulin secretion, as magnesium inadequacy is associated with β-cell dysfunction and insulin resistance, which are prominent hallmarks of diabetes mellitus (type 2) (Figure 3). In type 2 diabetes mellitus patients, the intracellular magnesium concentration is low, and an inverse association exists between the plasma level of magnesium and insulin resistance (5). A low intracellular magnesium concentration may impact subcellular insulin signaling activity to modify insulin sensitivity. In pancreatic β-cells, glucose metabolism is initiated by the conversion of glucose to glucose-6-phosphate, which is catalyzed by the enzyme glucokinase. This reaction is a crucial first step that eventually leads to an increase in intracellular ATP levels. The inadequacy of magnesium can impair the glucose metabolism process in pancreatic β-cells by reducing glucokinase activity due to limited Mg-ATP availability, disrupting the translocation of glucokinase from the nucleus to the cytoplasm and potentially altering the affinity of glucokinase for glucose, resulting in decreased glucose binding and reduced catalytic activity. Studies on patients with type 2 diabetes have shown that magnesium supplementation improves insulin sensitivity. There are dualistic interactions between magnesium metabolism and glucose metabolism, with magnesium being a cofactor of numerous enzymes involved in glucose metabolism that can also influence the bioactivities of insulin, while insulin stimulates magnesium uptake in insulin-sensitive tissues (5). Future prospective studies to minimize magnesium inadequacy before and during the appearance of the cluster of conditions associated with metabolic syndrome are needed to determine the potential benefits of magnesium on cardiometabolic risk in the general population.
